# Acute Aerobic Exercise-Induced Motor Priming Improves Piano Performance and Alters Motor Cortex Activation

**DOI:** 10.3389/fpsyg.2022.825322

**Published:** 2022-03-18

**Authors:** Terence Moriarty, Andrea Johnson, Molly Thomas, Colin Evers, Abi Auten, Kristina Cavey, Katie Dorman, Kelsey Bourbeau

**Affiliations:** ^1^Department of Kinesiology, University of Northern Iowa, Cedar Falls, IA, United States; ^2^School of Music, University of Northern Iowa, Cedar Falls, IA, United States

**Keywords:** aerobic exercise, high-intensity interval training, moderate-intensity training, motor skill performance, motor priming, piano, motor cortex, fNIRS

## Abstract

Acute aerobic exercise has been shown to improve fine motor skills and alter activation of the motor cortex (M1). The intensity of exercise may influence M1 activation, and further impact whole-body motor skill performance. The aims of the current study were to compare a whole-body motor skill *via* a piano task following moderate-intensity training (MIT) and high-intensity interval training (HIIT), and to determine if M1 activation is linked to any such changes in performance. Nine subjects (seven females and two males), aged 18 ± 1 years completed a control, MIT, and HIIT trial followed by administration of a piano performance task. M1 activation was evaluated by measuring oxyhemoglobin (O_2_Hb) and hemoglobin difference (Hbdiff) changes during post-exercise piano performance using functional near-infrared spectroscopy (fNIRS). The results indicate that piano performance scores were higher after the MIT trial, but not HIIT trial, compared to the control trial. A negative relationship was detected between heart rate during HIIT and post-HIIT piano scores. M1 activation (as measured by Hbdiff) was significantly increased after the HIIT trial. M1 activation was also positively associated with piano performance when exercise trials (HIIT + MIT) and all trials (HIIT + MIT + Control) were combined. We found that acute moderate-intensity exercise led to an improvement in complex motor skill performance while higher-intensity exercise increased M1 activation. These results demonstrate that moderate-intensity exercise can prime the nervous system for the acquisition of whole-body motor skills, suggesting that similar exercise protocols may be effective in improving the outcomes of other motor tasks performed during regular routines of daily life (e.g., sporting tasks, activities of daily living or rehabilitation). In addition, it appears that improvements in motor task performance may be driven by M1 activation. Our findings provide new mechanistic insight into the complex relationship between exercise intensity, M1 activation, and whole-body motor skill performance.

## Introduction

While it appears that acute aerobic exercise may improve laboratory-based simple motor task performance in humans (Sullivan et al., 2011; Mang et al., 2014, 2016; Statton et al., 2015; Perini et al., 2016; Snow et al., 2016), little is known regarding whether these exercise-induced improvements translate to motor skill performance during tasks of daily living (e.g., live musical performance). Participating in an acute bout of aerobic exercise pre- or post-motor skill practice may alter brain activation and impact skill performance and/or retention, a concept termed ‘exercise priming.’ Exercise priming may improve skill performance during motor skill acquisition (online learning) or may enhance motor memory consolidation *via* improved retention (offline learning; Wanner et al., 2020a). The extent to which acute aerobic exercise enhances motor skill performance may occur in an exercise-intensity dependent manner.

Several studies report that high intensity aerobic exercise may improve offline learning. For example, Roig et al. (2012), showed that a single bout of high-intensity interval training (HIIT; 3 bouts; 3-min at 200–315 W interspersed with 2-min at 50 W) performed on a cycle ergometer improved visuomotor accuracy-tracking task retention (24-h and 7 days post-acquisition). Interestingly, this was observed both when the exercise bout was performed before and when it was performed after motor skill practice (Roig et al., 2012). These findings are in agreement with several similar studies that also examined the impact of HIIT on offline learning in a variety of motor tasks including: continuous tracking with a joystick (Mang et al., 2014), serialized targeting (Mang et al., 2016), rotational visuomotor task (Ferrer-Uris et al., 2017), and a trigger task (Stavrinos and Coxon, 2017). In addition, evidence from a recent meta-analysis by Wanner et al. (2020a) also supports the motor priming effect of HIIT to enhance offline learning (SMD: 0.40, 95% CI: 0.05–0.75, *p* = 0.02). In contrast, no effect of acute exercise [HIIT or moderate-intensity training (MIT)] on general motor task online learning was detected by Wanner et al. (2020a) (SMD: 0.10; 95% CI: −0.08–0.29, *p* = 0.28). Subgroup analyses, however, revealed a significant effect of acute aerobic exercise on online learning of motor adaptation (but not motor skill learning) tasks. The researchers defined motor adaptation tasks as tasks that consist of a change in movement or performance driven by an environmental perturbation or change, compared to motor skill learning tasks that were characterized as tasks in which the participant was required to repeatedly perform a movement without external perturbations to learn a new skill (Wanner et al., 2020a).

While several studies report no positive effect of HIIT on online learning (Roig et al., 2012; Mang et al., 2016) limited evidence suggests that online learning may be enhanced after an acute bout of MIT. For example, Statton et al. (2015) observed a positive online learning effect following an acute bout of moderate-intensity aerobic exercise (30-min of running at 65–85% age-predicted HR_max_). Specifically, this task involved participants isometrically squeezing a force transducer between the thumb and index finger of the dominant hand to control the speed and accuracy of a cursor on a computer monitor. Additionally, Chartrand et al. (2015) report improved online learning of a laparoscopic surgical task following MIT (20-min of running at 60% VO_2max_). At first glance, these results suggest that HIIT may be optimal for offline learning while MIT may be optimal for online learning. However, studies comparing HIIT to MIT report no between group differences for both offline and online learning (Baird et al., 2018; Wanner et al., 2020b). It is important to note that the majority of literature examining the impact of exercise on motor skill acquisition and retention focuses on laboratory-based simple motor tasks. While these tasks play an important role in understanding motor learning, they may not capture the complex nature of motor tasks performed during the regular activities of daily living, such as playing a musical instrument (Ingram and Wolpert, 2011). Since complex tasks are associated with higher cognitive load (Van Merriënboer et al., 2006), and repetitive aerobic exercise (e.g., cycling in place) likely requires less cognitive engagement (Best, 2010), we speculate that in comparison to a more simple/traditional task, acute repetitive aerobic exercise may spare cognitive load and therefore optimize resources during the performance of a challenging post-exercise task. To our knowledge, only one study has explored the relationship between exercise priming and motor skill when motor skill performance is assessed as learning to play a musical instrument. Swarbrick et al. (2020) examined whether HIIT (3 bouts; 3-min at 90% watt max interspersed with 2-min at 60% watt max) or low-intensity interval training (LIIT; 3 bouts; 3-min at 12% watt max interspersed with 2-min at 8% watt max), when performed after practicing a piano melody, would impact retention and transfer of learning (to a new piano melody). No between group differences (HIIT vs. LIIT) were observed for online or offline learning however the HIIT group demonstrated modestly better transfer of learning when compared to the LIIT group (Swarbrick et al., 2020).

In addition to exploring whether exercise-intensity modulates motor skill performance, considerable efforts have been made to investigate underlying mechanisms that may explain the relationship between exercise priming and motor task performance. One mechanism that has been largely unexplored but may explain the improvement in downstream motor skill performance is an exercise-induced increase in activation of the primary motor cortex (M1). During voluntary movements, M1 excitability increases, making it a common site of examination for researchers investigating neuromotor behavior (Nishiyori et al., 2016). Neural activity is closely tied to cerebral blood flow where an increase in activation leads to an increase in regional blood flow, in an effort to meet the metabolic demand of the active cerebral tissue (Scholkmann and Wolf, 2012). Thus, functional near-infrared spectroscopy (fNIRS), which monitors changes in oxy- and deoxyhemoglobin (indicative of blood flow changes), has recently been used as a tool to indirectly assess cerebral activation (Scholkmann and Wolf, 2012). Although evidence suggests that M1 oxygenation increases following acute aerobic exercise (Tsubaki et al., 2018), less is known regarding whether this translates to changes in motor skill performance. In addition, the relationship changes in M1 activation following acute aerobic exercise of different intensities (MIT vs. HIIT) and subsequent impact on the performance of motor tasks representative of activities of daily living remains to be elucidated. It is important to note that intent of the current study was not to assess the effects of acute aerobic exercise on motor skill learning but rather post-exercise motor skill performance. Therefore, the aims of the present investigation were twofold: (1) Examine whether an acute bout of MIT or HIIT differentially impact whole-body motor skill performance (as assessed using a piano performance task) and (2) Explore whether performance on the piano performance task is related to M1 activation (as assessed using fNIRS) or markers of exercise intensity [blood lactate (Bla^−^), heart rate (HR), rating of perceived exertion (RPE)]. Since it appears performing acute MIT may reduce cognitive load and be beneficial for online learning involving simple motor tasks, we hypothesized that MIT, but not HIIT, would improve piano performance. In addition, and in light of the fact that previous research has found increased M1 activation following acute aerobic exercise, it was hypothesized that M1 activation would be greater following both acute HIIT and MIT. Finally, given that the relationship between M1 activation and motor performance remains to be elucidated, we hypothesized that the increase in M1 activation would not be related to changes in piano performance.

## Materials and Methods

### Participants

All participants were undergraduate students at the University of Northern Iowa (UNI) enrolled in their second semester of MUS APPL 1470 Group Piano for Music Majors having successfully passed their first semester with a C grade or higher. A total of 9 participants (male = 2, female = 7) volunteered to take part in this study (Table 1). Prior to signing the consent form, all risks, benefits, and procedures were outlined and a health history questionnaire was filled out. Participants reported no known cardiovascular, pulmonary, or metabolic disorders. In addition, participants had no known history of psychiatric illness or neurological brain disease and were not excluded on the basis of a history of depression, provided it had been effectively treated. All study procedures were completed in the Exercise Physiology and Music Performance Laboratory at UNI under similar environmental conditions and at the same time of day (± 2 h). The protocol was approved by the UNI Institutional Review Board.

**Table 1:** Subject characteristics.

**Characteristic**	***N* = 9 (2 males and 7 females)**
Age (years)	18 ± 1
Height (cm)	169.3 ± 7.5
Weight (kg)	73.8 ± 13.9
Body mass index (kg/m^2^)	25.9 ± 5.8
Body fat (%)	30.1 ± 14.2
VO_2max_ (ml/kg/min)	32.4 ± 9.7
Months of musical training	32.6 ± 38.5
GPA	3.63 ± 0.27
IPAQ (MET-mins/week)	2,093 ± 1,660

*Mean ± SD. cm, centimeters; GPA, grade point average; IPAQ, International Physical Activity Questionnaire (MET-mins/week). kg, kilograms; kg/m^2^, kilograms per square meters; VO_2max_, maximum volume of oxygen consumed (ml/kg/min)*.

### Study Design

**Figure 1:**
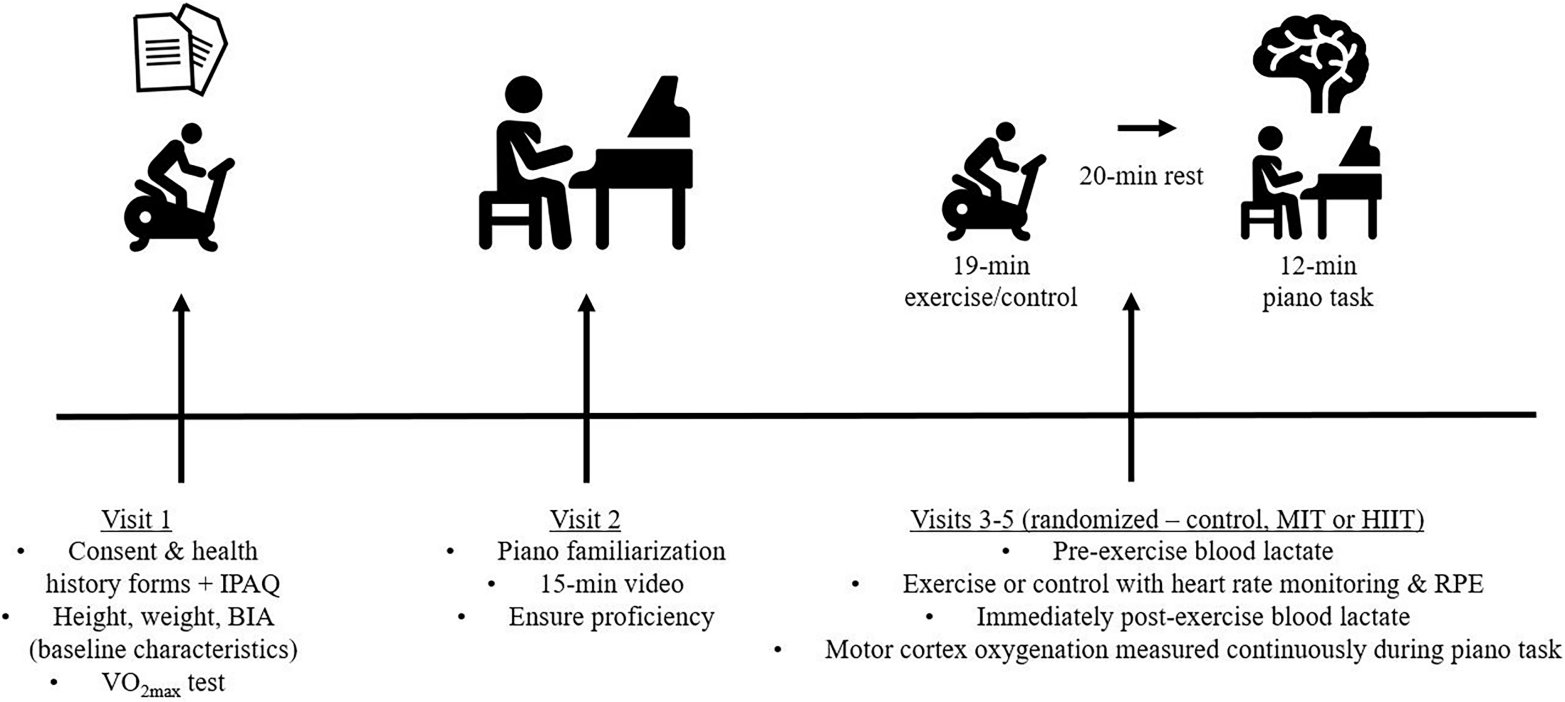
Testing procedures. BIA, bioelectrical impedance analysis; HIIT, high-intensity interval training; IPAQ, International Physical Activity Questionnaire; MIT, moderate-intensity interval training; VO_2max_, maximal oxygen consumption test.

Each participant completed baseline testing and a piano familiarization trial followed by three trials in a randomized fashion (MIT, HIIT, and control). All trials were separated by at least 48 h. Baseline measures included a maximal oxygen consumption test (VO_2max_), body composition *via* bioelectrical impedance analysis (BIA), and International Physical Activity Questionnaire (IPAQ). Upon arrival for the piano familiarization participants were asked to view a pre-recorded video outlining the required piano tasks to be evaluated. Participants met in small groups and were given the opportunity to engage with material similar to the piano tasks encountered in the experimental trials. The piano classroom was outfitted with 13 Yamaha Clavinova electric pianos, allowing participants to join simultaneously on different instruments. The participants were given timed opportunities to practice musical examples highlighted in the video recording. Both exercise trials began with the participant being fitted with a heart rate monitor while sitting on a cycle ergometer and measurements of resting HR and Bla^−^ were collected. Both exercise conditions were 19 min in total with measurements of HR and RPE performed throughout each trial. Blood lactate was also collected prior to and immediately following each exercise trial and prior to the control trial. Following completion of the exercise portion of the trial or control (time matched), participants completed a 20-min rest period before the piano performance task. The 20-min time period prior to piano performance was chosen as it has been used by previous researchers with positive results on cognitive function and motor task performance (Chang et al., 2012; Kendall et al., 2020). During this time, participants were instructed to refrain from using electronic devices and to limit communication with any research team members present in the room. Finally, the participant was fitted with the fNIRS headcap and the piano task was administered. An outline of the study design is shown in Figure 1.

### Baseline Measures

Following approval and signature of the consent form, the IPAQ was administered and each participant’s height (nearest 0.1 cm) and weight (nearest 0.1 kg) were measured using a stadiometer and scale, respectively. These measures were used to calculate each subject’s body mass index (BMI). In addition, body fat percentage was determined using a bioelectrical impedance analysis BIA device (InBody 720, Cerritos, CA, United States). The same technician performed the BIA test for all participants.

Following initial measurements, the subject performed a VO_2max_ test on an electronically-braked cycle ergometer (Lode, Groningen, The Netherlands) to determine cardiovascular fitness (VO_2max_). Following a brief, self-select warm-up, participants were connected to a metabolic cart (Parvomedics, Sandy, UT, United States) in order to measure oxygen consumption (VO_2_) and carbon dioxide production (VCO_2_). During the test, participants were asked to maintain 70–80 revolutions per minute (RPM). Heart rate was monitored continuously using a Polar^™^ HR monitor (V800, Polar Electro Inc., Woodbury, NY, United States) and workload was increased every 3 or 4 s (20-watt ramp for males beginning at 40 watts and 15-watt ramp for females beginning at 30 watts) until cadence dropped below 60 RPM despite verbal encouragement or subjects reached volitional fatigue (Moriarty et al., 2019). That said, participants were asked to stop and inform the researcher if they experienced any chest pain, dizziness, or faintness. VO_2max_ required two of the following four criteria to be met: respiratory exchange ratio (RER) > 1.15, within ±10 bpm of age-predicted maximal HR, VO_2_ plateau of ≤150 ml·min^−1^, or RPE > 17. VO_2max_ was determined by the highest value achieved using an 11-breath rolling average. Participants’ workload in watts, in combination with %VO_2max_, were used to calculate appropriate high or moderate intensity levels for each of the participant’s assigned exercise protocols.

### Piano Familiarization and Task

Prior to completing the randomized trials, participants watched a 15-min instructional video and were required to demonstrate proficiency and comprehension of the piano tasks. Participants were familiarized with the required tasks to be performed during the experimental and randomized trials and allowed to practice the tasks in a similar fashion. Each randomized trial included variations in each of the following tasks: two sight read musical examples with treble and bass clef notation, transposition of written musical examples, one sight read open score example, and performance of scales and arpeggiated vocal warm-up exercises. In both the familiarization and randomized trials participants were given 30 s to silently review each sight-read musical example, with instructions to write any helpful indications onto the score. Participants were given an additional 30 s to practice the example before performing it in full and being graded by three piano teachers for a score out of 100. All three teachers were blinded to the participants’ randomized trial.

### Control and Exercise Protocols

#### MIT and HIIT Trials

Both exercise trials (MIT and HIIT) were 19 min in total duration and were based upon the participant’s VO_2max_ from the first trial. During the MIT trial, each subject completed a 2-min warm up at 30% intensity (workload in watts at 30%VO_2max_) followed by 15 min of continuous cycling at 50% intensity and a cool down at 30% intensity for another 2 min. The HIIT trial consisted of identical warm-up and cool down phases. The main phase during this trial consisted of 1-min cycling bouts at 85–95% intensity (workload in watts at 85–95%VO_2max_) followed by 2-min of recovery at 40% intensity. Heart rate and RPE were recorded at the end of the warm-up and every 3 min thereafter during both exercise trials. The participants were asked to rate the intensity of exercise, by using the Borg scale (6–20). A Bla^−^ measurement was also taken before and after each of the randomized trials. Motor cortex activation was continuously evaluated using fNIRS (Octamon +, Artinis Medical Systems, Elst, The Netherlands – 8-channel setup) during the piano performance that lasted approximately 12 min.

#### Control Trial

Participants in the control trial were required to sit quietly for the same period of time as the MIT and HIIT exercise bouts. Following 19 min of rest, these participants also completed the same 20-min rest period as the exercise trials before beginning the piano performance task. During this time, subjects were instructed to stay off of all electronic devices and limit communication with any member of the research team in the room.

### Functional Near Infrared Spectroscopy Recording

**Figure 2:**
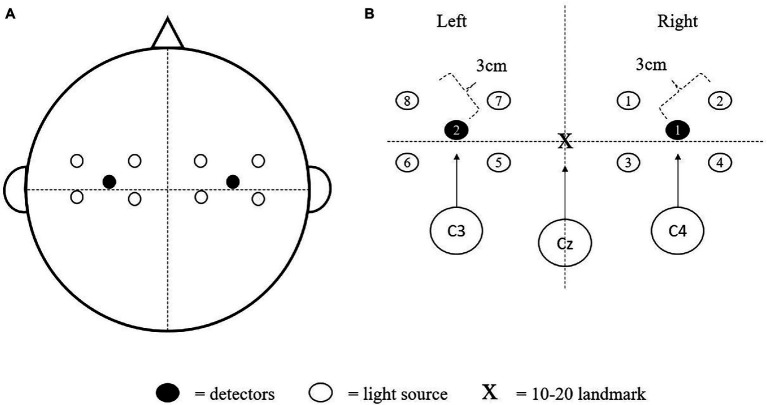
Octamon + light source and detector configuration covering the bilateral motor cortex. In **(A)** shows a superior view of the orientation of the setup on the head; and **(B)** the position of the eight light sources and two detectors relative to the international electroencephalogram 10–20 landmarks (C3-Cz-C4 line).

All fNIRS recordings during the piano performance task were performed using a noninvasive, portable, 8-channel dual wave-length (760 and 850 nm) optical system (OctaMon+, Artinis Medical Systems, Elst, The Netherlands) at a sampling rate of 10 Hz. This device is wireless and capable of measuring continuous brain oxygenation during cognitive and motor tasks (Lin et al., 2013; Byun et al., 2014). The setup consisted of 4 light sources and 1 detector over the left M1 and the same over the right motor cortex (8 × 2 configuration; see Figure 2). The light sources and detectors were connected to a 5 mm neoprene headcap, which held them at a distance of approximately 30 mm apart. The approximate location of the light sources were on the left and right hemispheres with the two detectors slightly forward of the C3-C4 area and center of the head aligned with the vertex (Cz), based on the modified international electroencephalogram (EEG) 10–20 system (Koenraadt et al., 2012; Blokland et al., 2013; Nishiyori et al., 2016). The M1 expands laterally from the central midline towards the ears (Krogh, 2014), has been identified by EEG C3 and C4 landmarks (Rich and Gillick, 2019), and has also been shown to be activated during hand movements (Koenraadt et al., 2012, 2013). Changes in oxyhemoglobin (O_2_Hb) and hemoglobin difference (Hbdiff) were used as indicators of motor cortex oxygenation and activation, using the modified Beer–Lambert Law. Since O_2_Hb changes from baseline during periods of neural activity (Ferrari et al., 2011), it was used as a marker of oxygenation, while Hbdiff changes were also included as this measurement is a sensitive measure of oxygen extraction and has been shown to have a high correlation with arterial pressure and blood flow (Tsuji et al., 1998). The fNIRS data were acquired using Oxysoft version 3.2.72 × 64, filtered using a 0.1 Hz lowpass filter to eliminate any potential noise (e.g., HR, speaking, breathing), and then averaged and analyzed in IBM SPSS Statistics (version 25.0, Chicago, IL, United States).

### Whole Blood Lactate Measurement

A droplet of blood (approximately 1 μl) was collected from the earlobe *via* lancet puncture in order to measure Bla^−^ pre and immediately post both exercise trials and prior to the control trial. A handheld analyzer device was used to analyze the Bla^−^ content from the droplet of blood (Nova Biomedical, Waltham, MA, United States). All samples were collected in duplicate and averaged for analysis.

### Data Analysis

All results are expressed as means ± SD. Sample size was determined based on *a priori* calculation with power set to 0.80 and alpha level of 0.05 (G^*^Power, Dusseldorf, Germany). The criteria for selected studies used for the calculation were acute exercise bouts and changes in motor skill performance (Hübner et al., 2018; Chen and Ringenbach, 2019). The estimated effect size was 0.58, which estimated a minimum of seven participants to detect a difference in the motor task given the chosen analyses. Two separate one-way ANOVAs with repeated measures were used to analyze changes in the M1 and differences in piano performance scores between trials (control, MIT, HIIT). A one-way ANOVA was also used to analyze changes in markers of exercise intensity (HR, RPE, Bla^−^, %HRmax). *Post hoc* Tukey analysis was performed when main effects were detected. Partial eta squares $\left(\eta_{p}^{2}\right)$ , a measure of effect size, was used for the magnitude of the mean effect size, interpreted as: low <0.04, medium ≥0.04 to <0.36, and large >0.36 (Cohen, 1988). Pearson correlation analyses were also performed to evaluate relationships between various exercise intensity markers (e.g., average HR, %HRmax, Bla^−^, and RPE) and piano performance scores, and between piano outcomes and M1 activation *via* fNIRS. Intraclass correlation (ICC) estimates and their 95% confidence intervals (CI) were calculated for the scoring of the piano performance task and based on a mean-rating (*k* = 3), absolute agreement, 2-way mixed-effects model. Under such conditions, the following categories were established: < 0.5 = poor, 0.5–0.75 = moderate, 0.75–0.9 = good, and > 0.9 = excellent (Portney and Watkins, 2009). Data were analyzed using IBM SPSS Statistics (version 25.0, Chicago, IL, United States).

## Results

### Demographics and Trial Parameters

Participant age, height, weight, BMI, body composition, maximal oxygen consumption, months of musical training, major GPA, and minutes of physical activity per week are shown in Table 1. Average male BMI was 21.3 kg/m^2^ and classified as “normal,” while average female BMI was 27.3 kg/m^2^ and classified as “overweight” (American College of Sports Medicine, 2018). Male’s aerobic fitness was 47.2 ml/kg/min and classified as “good,” while female’s aerobic fitness was 28.2 ml/kg/min and classified as “fair” (American College of Sports Medicine, 2018). In terms of the IPAQ, seven out of the nine participants scored in the “minimally active” category, one female scored “inactive” and one female scored “more active.” It is important to note that although the two male subjects scored in the “minimally active” category they reported 2974.5 and 2916.5 MET-mins/week, respectively, which was just below the cut-off of 3,000 MET-mins/week to classify as “more active.” Finally, average male body composition (11.6% body fat) was classified as “good” and average female body composition (35.4% body fat) was classified as “very poor” (American College of Sports Medicine, 2018). Based on the BMI, fitness, and body composition data, we conclude that male participants in the current investigation were deemed to be healthy college-aged participants while female participants were deemed to be less physically fit and unhealthy in terms of body composition.

A significant main effect (*p* < 0.01) was observed between trials for all exercise intensity indicators [HR: *F*_(2,16)_ = 204.305, $\eta_{p}^{2}$ = 0.962, large; RPE: *F*_(2,16)_ = 214.643, $\eta_{p}^{2}\;$ = 0.963, large; Bla^−^: *F*_(2,16)_ = 76.917, $\eta_{p}^{2}$ = 0.906, large; %HRmax: *F*_(2,16)_ = 216.851, $\eta_{p}^{2}$ = 0.964, large]. Participants completed all study trials, including control, MIT, and HIIT in randomized order. As expected, exercise intensity indicators were higher (*p* < 0.01) in the HIIT compared to the MIT and control, and also higher (*p* < 0.01) in the MIT compared to the control (see Table 2).

**Table 2:** Exercise intensity variables.

**Measurement**	**HIIT**	**MIT**	**Control**
Duration (min)	19	19	19
HR (bpm)	171 ± 8.1^**^^	146 ± 11.2^**^	84 ± 12.1
%HRmax	89 ± 4%^**^ ^	76 ± 7%^**^	44 ± 7%
Bla^−^ (mmol/L)	5.1 ± 1.1^**^^	2.7 ± 1.0^**^	1.0 ± 0.1
RPE	15.4 ± 1.2^**^^	12.4 ± 1.2^**^	6 ± 0

*Mean ± SD for each trial (*N* = 9). Bla^−^, blood lactate; bpm, beats per minute; HIIT, high-intensity interval training; HR, heart rate; min, minutes; MIT, moderate-intensity training. mmol/L, millimoles per liter; RPE, rating of perceived exertion*.

** *Statistically higher than control, *p* < 0.01*.

^ *Statistically higher than MIT, *p* < 0.01*.

### Post-exercise Piano Performance

The ICC among raters was excellent for the MIT (ICC = 0.942, 95% CI = 0.753–0.987), excellent for the HIIT (ICC = 0.962, 95% CI = 0.863–0.991) and good for the control trial (ICC = 0.895, 95% CI = 0.686–0.974). A significant main effect was detected for post-exercise piano performance [*F*_(2,16)_ = 5.74, *p* < 0.05, $\eta_{p}^{2}$ = 0.418, large]. *Post hoc* testing revealed significantly higher piano scores following MIT compared with control (89.7 ± 7.8 vs. 79.6 ± 13.5, *p* < 0.05) but not HIIT (87.6 ± 9.1, *p* > 0.05).

### Motor Cortex Oxygenation During Piano Performance

**Figure 3:**
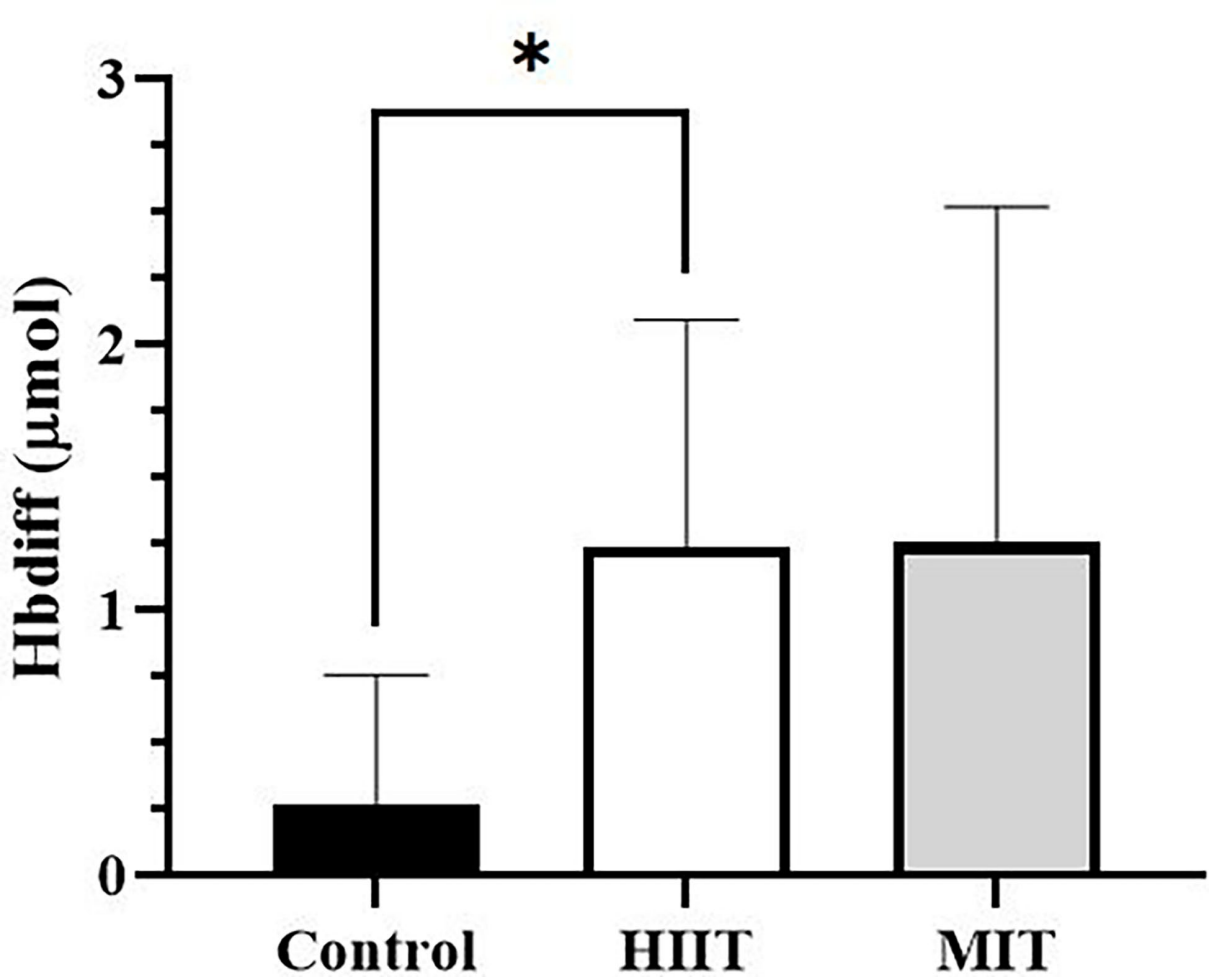
Motor cortex hemoglobin difference (Hbdiff) levels after control and exercise conditions. An increase in activation as reported by Hbdiff was higher after high-intensity interval training (HIIT) compared to the control trial. ^*^Significantly higher than control, *p* < 0.05. MIT, moderate-intensity training. *N* = 6.

The fNIRS measurement was recorded during all piano performances (20 min after the control (seated rest), MIT and HIIT trials). A significant main effect was detected for Hbdiff [*F*_(2,10)_ = 5.71, *p* < 0.05, $\eta_{p}^{2}$ = 0.533, large] during the piano performance. *Post hoc* testing revealed significantly higher Hbdiff during the piano task after HIIT compared with control (1.24 ± 0.85 vs. 0.27 ± 0.49 μmol, *p* < 0.05, Figure 3). No main effect was detected for O_2_Hb using the fNIRS.

The fNIRS data of three subjects were unusable and were removed from the analysis. Thus, the data of the remaining six subjects were analyzed and reported as preliminary data.

### Piano Performance Scores and M1 Activation

**Figure 4:**
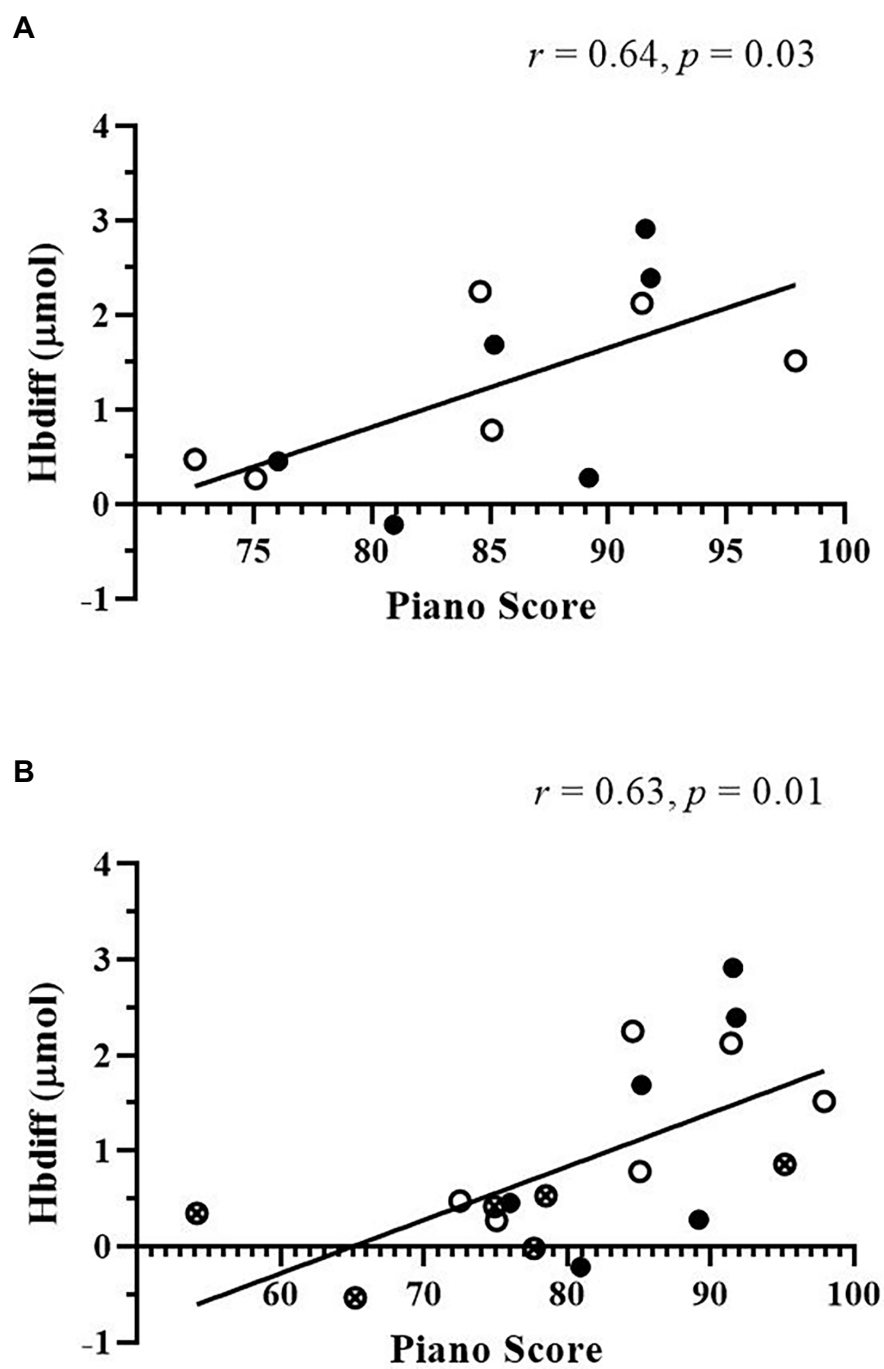
Relationship between motor cortex hemoglobin difference (Hbdiff) and piano scores. Significant positive relationships were detected when combining all exercise data **(A)** and with the addition of the control data **(B)**. High-intensity interval training data. Moderate-intensity training data. Control data. *N* = 12 **(A)** and *N* = 18 **(B)**. White dot = high-intensity interval training data. Black dot = moderate-intensity training data. Dot with x = control data

A correlational analysis was used to evaluate the relationship between piano performance scores and M1 activation for each trial. No associations were found between M1 activation and piano scores after each exercise or control trial. However, when exercise data was combined together (MIT + HIIT), a significant positive relationship was detected between Hbdiff and piano scores (*r* = 0.64, *p* = 0.03, Figure 4A). In addition, when the control trial was added (MIT + HIIT+control), this relationship was kept (*r* = 0.63, *p* = 0.01, Figure 4B). This indicates greater oxygen extraction (Hbdiff) post-exercise and post-control was associated with higher piano performance scores. No relationships existed between O_2_Hb and piano scores.

### Exercise Intensity Markers and Piano Performance Scores

**Figure 5:**
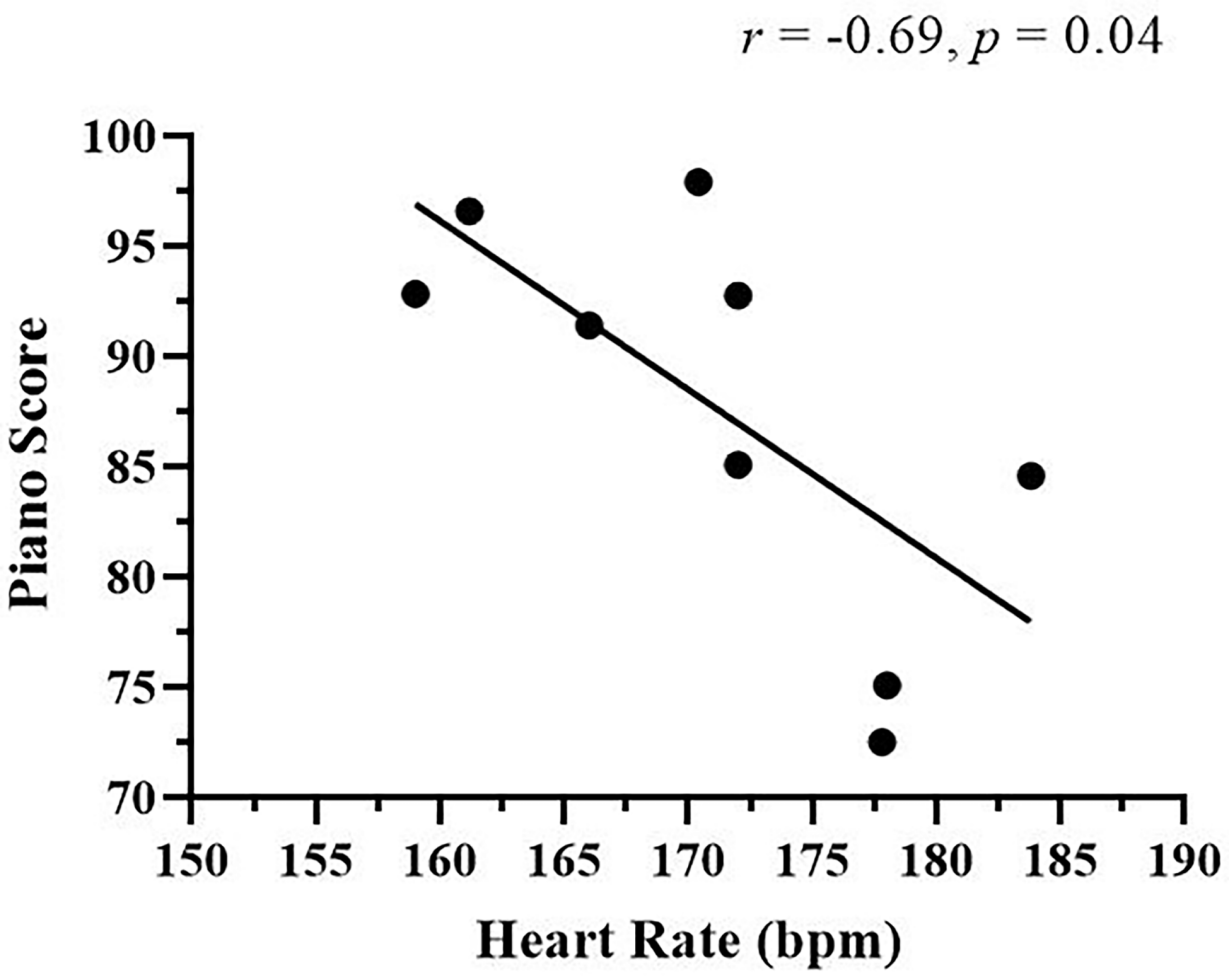
Relationship between high-intensity interval training (HIIT) heart rate and post-HIIT piano scores. A significant negative relationship was detected. bpm, beats per minute. *N* = 9.

Correlation analyses were performed to determine if associations existed between exercise intensity parameters (HR, %HRmax, Bla^−^, and RPE) and piano performance scores. Heart rate during the HIIT trial had a significant negative relationship with post-HIIT piano performance (*r* = −0.69, *p* = 0.04; Figure 5). This indicates that higher intensity cycling was associated with worsening piano task performance. No relationships existed between %HRmax, Bla^−^, RPE and piano scores.

## Discussion

The aim of the present study was to investigate whether performing an acute bout of moderate (MIT) or high-intensity (HIIT) aerobic exercise prior to a piano motor task impacts piano task performance. Further, we aimed to explore whether piano task performance was related to either post-exercise M1 activation (as measured by Hbdiff) or markers of exercise intensity (e.g., HR, %HRmax, Bla^−^, RPE). Findings from this study indicate that MIT, but not HIIT led to superior piano task performance, when compared to control. Further, preliminary fNIRS data suggest that M1 activation was significantly higher during piano task performance following HIIT but not MIT when compared to control. Taken together, these results propose that the HIIT-induced increase in M1 activation did not translate to better piano task performance. Interestingly, however, when examining the relationship between M1 activation and piano performance in combined groups (MIT + HIIT), a positive association was observed where increased M1 activation was related to better piano task performance. This significant relationship remained when all trials (MIT + HIIT + Control) were combined, suggesting that M1 activation is related to piano task performance but perhaps not as a result of exercise intensity. In fact, a negative association between HR during HIIT and piano performance was observed in the present study. Specifically, the higher the average HR during HIIT, the worse the piano performance score. Thus, if the goal of exercise priming is to enhance the performance of a whole-body motor skill, implementing moderate intensity aerobic exercise prior to motor skill performance may be optimal.

Recent research has made it increasingly evident that there is a complex interaction between exercise intensity and motor task performance (Opie and Semmler, 2019; Wanner et al., 2020a). The findings from the present study are in agreement with previous reports that MIT, when performed prior to a motor task, may optimize improvements in online learning (Chartrand et al., 2015; Statton et al., 2015; Snow et al., 2016; Opie and Semmler, 2019). Our results further align with studies that report no effect of HIIT on post-exercise online learning (Roig et al., 2012; Mang et al., 2016). One proposed mechanism that may explain why MIT, but not HIIT, improved piano performance is related to the cognitive component related to online learning. Piano performance involves motor and cognitive functions simultaneously. Such functions required in the performance of a musical example by sight include: a multi-faceted activity involving eye-hand span (Rayner and Pollatsek, 1997), executive function (Kim and Yoo, 2019), working memory (Herrero and Carriedo, 2019), visual processing (Arthur et al., 2021), and the transformation of visual information to motor output (Rosemann et al., 2016). Specifically, MIT-induced changes in executive function may impact motor task performance during online learning (McMorris et al., 2015; Snow et al., 2016; Stoykov et al., 2017). For example, Baird et al. (2018) suggested that acute aerobic exercise particularly improves the cognitive portion of the performance and not the motor aspects. This is further supported by Kamijo et al. (2004) showing that information processing is closely related to the intensity of exercise, with moderate-intensity exercise producing optimal attentional resources. Since these participants were experienced pianists, our results indicate that these tasks were cognitively demanding even for the experienced players. Therefore, the tasks could not be completed automatically and results may suggest that the moderate-intensity exercise optimized attentional resources during the post-exercise piano performance. In contrast to MIT, HIIT may have induced greater post-exercise fatigue or taxing of cognitive resources. Post-exercise fatigue may have counteracted the beneficial effects of exercise on motor skill performance. While the results of the current study may apply to individuals of “fair-good” aerobic fitness, it is important to note that fitness level may have an impact upon the findings. Previous research has demonstrated that although high-fit individuals have greater cognitive performance when compared to low-fit individuals at baseline, low-fit individuals might be more responsive to acute exercise and benefit more from the exercise (Li et al., 2019). Therefore, acute aerobic exercise performed prior to motor skill performance may not be as beneficial for those of higher fitness levels and this should be considered in future studies. In addition, a mechanistic impact of M1 activation may further explain why HIIT did not enhance post-exercise motor skill performance in the present study. As mentioned, our preliminary data suggest increased M1 activation following HIIT. These results are in line with Brümmer et al. (2011) who found that M1 activity was intensified with increasing exercise intensity. In an effort to support increased M1 activation, CBF may be redirected from cognitive related areas of the brain (e.g., prefrontal cortex) towards the M1 region in an effort to sustain the physical demand of the intense exercise bout, potentially sacrificing motor performance due to interference with necessary cognitive processes. It is important to note, however, that a significant positive correlation was observed in the present study between piano task performance and M1 activation when MIT + HIIT and MIT + HIIT + Control trials were combined. These results may suggest that although M1 activation plays an important role in post-exercise motor performance, this may not be the result of exercise intensity. Further, an optimal level of M1 activation may exist that translates to improvements in online learning (Neva et al., 2019). This theory, however, warrants further exploration.

Intensity-dependent physiological changes during and post-acute aerobic exercise may further explain the role of motor priming for whole-body motor skill performance. In particular, aerobic exercise is known to influence blood flow and activation to specific cortical areas. The association between exercise intensity markers and piano performance scores revealed a negative correlation between HR during HIIT and subsequent piano task performance. This result suggests that, in our sample, an increase in HR during the higher-intensity exercise bout was linked with worsening piano performance. Similar observations have been made in young male soccer players and young healthy males and females in response to acute high-intensity exercise (McMorris and Rayment, 2007; Stavrinos and Coxon, 2017). For example, McMorris and Rayment (2007) found that short duration high-intensity sprinting had a negative effect on soccer passing accuracy which requires both perceptual judgement and motor control.

### Limitations

Although not specifically studied in the current investigation, it is important to note that high-intensity aerobic exercise may be warranted to drive longer lasting improvements in complex motor skill retention. Therefore, MIT may promote a positive physiological stimulus to impact post-exercise motor skill performance but higher intensity exercise may be needed for longer-term adaptations related to learning. That said, it is possible that when motor practice and MIT are paired over multiple sessions there is a beneficial and/or additive effect on offline learning (Statton et al., 2015). Several limitations exist in the current study. First, the small sample size and recruitment from one university may limit external validity. To fully understand the impact of acute exercise on piano performance, a larger sample size with a more balanced proportion of males and females, similar level of background training, and varying cultural and ethnic backgrounds is warranted. Specific to the sample size, the piano performance scores for HIIT and MIT were very similar and with a larger sample size it is highly probable the HIIT condition would have also improved piano performance. Similarly, the mean value for M1 activation during the MIT condition was slightly higher than the HIIT condition, but with higher variability. Therefore, with a larger sample size, it is also probable that MIT M1 activation would have been significantly higher than the control trial. It should also be noted that these fNIRS data are preliminary and results need to be interpreted with caution. Second, three subjects fNIRS data had to be excluded since no significant hemodynamic response was able to be found. Previous research has shown that non-responders are often reported in fNIRS studies due to larger skull thickness, darker hair pigmentation or greater hair density, or varying skull-to-cortex distances (McIntosh et al., 2010; Haeussinger et al., 2011). Finally, since M1 activity was assessed using a superficial measurement tool over a limited brain region, examining more global brain regions and/or using a more invasive method to do so (e.g., fMRI) may provide additional insight into this response.

In conclusion, exercise intensity influenced piano performance, with improved performance after MIT. In addition, exercise intensity influenced motor cortical activation, with increased M1 activation following HIIT. That said, a negative correlation between average HR during the HIIT trial and post-HIIT piano performance score was found. Therefore, fatiguing exercise may be detrimental to complex, whole-body motor tasks performed 20 min after exercise while the application of moderate-intensity exercise priming to improve the performance skill level may extend to other sporting tasks (e.g., painting, writing, wood working) or clinical populations (e.g., Stroke, Parkinson’s, Alzheimer’s). The positive relationship between piano scores and M1 activation following exercise or a period of rest also provides additional insight into potential future research opportunities investigating mechanisms of such a relationship. Future research is warranted to further extend our understanding of this preliminary fNIRS data. The results also highlight the importance of studying the short-term and longer-term effects of exercise intensity in modulating neurophysiological mechanisms that are responsible for whole-body motor task performance improvements.

## Data Availability Statement

The raw data supporting the conclusions of this article will be made available by the authors, without undue reservation.

## Ethics Statement

The studies involving human participants were reviewed and approved by University of Northern Iowa Institutional Review Board. The patients/participants provided their written informed consent to participate in this study.

## Author Contributions

TM, AJ, and KB conceptualized the experiment and wrote the original draft of the manuscript. TM, AJ, MT, CE, AA, KC, and KD collected the data. TM and KB processed and conducted statistical analyses. TM, AJ, MT, CE, KB, AA, KC, and KD edited and approved the final manuscript. All authors contributed to the manuscript and approved the submitted version.

## Funding

This work was supported by the University of Northern Iowa.

## Conflict of Interest

The authors declare that the research was conducted in the absence of any commercial or financial relationships that could be construed as a potential conflict of interest.

## Publisher’s Note

All claims expressed in this article are solely those of the authors and do not necessarily represent those of their affiliated organizations, or those of the publisher, the editors and the reviewers. Any product that may be evaluated in this article, or claim that may be made by its manufacturer, is not guaranteed or endorsed by the publisher.
